# Association of electroencephalogram epileptiform discharges during cardiac surgery with postoperative delirium: An observational study

**DOI:** 10.3389/fsurg.2022.900122

**Published:** 2022-09-06

**Authors:** Na Li, Xing Liu, Yuhua Gao, Lingzi Yin, Wanli Zhao, Rongxing Ma, Xinli Ni

**Affiliations:** ^1^ Department of Anaesthesiology, General Hospital of Ningxia Medical University, Yinchuan, China; ^2^ Department of Neuroelectrophysiology, General Hospital of Ningxia Medical University, Yinchuan, China

**Keywords:** electroencephalography, epileptiform discharges, cardiac surgery, postoperative delirium, cardiopulmonary bypass

## Abstract

**Background:**

Delirium is a frequent and serious complication following cardiac surgery involving cardiopulmonary bypass (CPB). Electroencephalography reflects the electrical activity of the cerebral cortex. The impact of electroencephalographic epileptiform discharges during cardiac surgery on postoperative delirium remains unclear. This study was designed to investigate the relationship between intraoperative epileptiform discharges and postoperative delirium in patients undergoing cardiac surgery.

**Methods:**

A total of 76 patients who underwent cardiac surgery under CPB were included. The baseline cognitive status was measured before surgery. Electroencephalograms were monitored continuously from entry into the operating room to the end of surgery. The presence of delirium was assessed through the Confusion Assessment Method or the Confusion Assessment Method for the Intensive Care Unit on the first 3 days after surgery. Univariate and multivariate logistic regression analyses were performed to evaluate the association between epileptiform discharges and delirium.

**Results:**

Delirium occurred in 31% of patients and epileptiform discharges were present in 26% of patients in the study. Patients with delirium had a higher incidence of epileptiform discharges (52.63% vs. 13.95%, *P* < 0.001) and longer durations of anesthesia and CPB (*P* = 0.023 and *P* = 0.015, respectively). In addition, patients with delirium had a longer length of hospital stay and a higher incidence of postoperative complications. Multivariate logistic regression analysis showed that age and epileptiform discharges were significantly associated with the incidence of postoperative delirium [odds ratio, 4.75 (1.26–17.92), *P* = 0.022; 5.00 (1.34–18.74), *P* = 0.017, respectively].

**Conclusions:**

Postoperative delirium is significantly related to the occurrence of epileptiform discharges during cardiac surgery.

## Introduction

Delirium is a type of acute brain dysfunction associated with changes in consciousness, attention, and cognitive function; it is common after cardiac surgery (1, 2). Delirium can increase hospitalization costs and length of stay (LOS), can reduce the quality of life, and is closely associated with early postoperative cognitive dysfunction (3–6). Notably, one of the pathophysiological mechanisms of delirium is neurotransmitter imbalance, which can lead to changes in electroencephalogram (EEG) patterns (7, 8). A study has found that adults who undergo cardiac surgery utilizing cardiopulmonary bypass (CPB) are more likely to experience seizures and increased operative mortality (9).

The epileptiform discharges on the EEG are a good indicator of the abnormal excitability of the brain; they are associated with neurocognitive decline (10), Alzheimer’s disease (11), hypoxic encephalopathy after cardiac arrest (12), and autism (13). Therefore, epileptiform discharges may provide a method for early identification of abnormal discharges of the brain. In our preliminary observations, we found that patients undergoing CPB demonstrate epileptiform discharges on the EEG during surgery. However, the relationship between epileptiform discharges and delirium is unclear.

This study was designed to investigate the relationship between epileptiform discharges on the EEG and postoperative delirium during cardiac surgery under CPB. It aimed to provide clinical evidence for the use of electroencephalography in patients with postoperative delirium.

## Materials and methods

### Patients

This single-center prospective observational study was performed at the General Hospital of Ningxia Medical University between July 2, 2020, and July 16, 2021. It was approved by the Ethics Committee of the General Hospital of Ningxia Medical University (KYLL-2021-358) and is registered at https://www.clinicaltrials.gov/ (NCT04943939). The study was performed according to the Declaration of Helsinki. All participants provided written informed consent before participating in the study.

A total of 76 patients having American Society of Anesthesiologists physical status III, who were aged >18 years and had corrected preoperative conditions, were scheduled for elective open-chamber cardiac valve reconstruction or replacement with cardiopulmonary bypass, and admitted to the cardiac surgical care unit after surgery were enrolled in this prospective observational study. Those receiving off-pump cardiac surgery; undergoing surgery for correction of congenital heart disease; having a history of stroke, schizophrenia, depression, epilepsy, dementia, and drug addiction; unable to communicate due to language impairment or significant hearing or visual impairment; having severe liver dysfunction or severe renal insufficiency requiring preoperative renal replacement therapy; or having a history of intraoperative awareness or falls in the last 6 months were excluded. Patients who withdrew consent and refused further participation were excluded from the study.

### Perioperative management

Patients received standard monitoring, including electrocardiography, pulse oximetry, and continuous monitoring of radial arterial blood pressure, nasopharyngeal temperature, end-tidal CO_2_, and urine output. Anticholinergic drugs such as scopolamine were strictly prohibited during the study period. Atropine was only used to treat bradycardia, and midazolam was not used as an anxiolytic. Etomidate or propofol, sufentanil, and rocuronium were used for induction of anesthesia. After tracheal intubation, anesthesia was maintained by continuous infusion of propofol, remifentanil, and rocuronium boluses according to clinical needs. The use of vasoactive drugs was personalized according to the patient’s condition. Propofol was administered with the depth of anesthesia adjusted based on the patient status index, which was generated by SedLine (Masimo Inc., United States). After the operation, patients were sent to the cardiac surgical care unit for further standardized treatment.

### EEG recording and analysis

The SedLine monitor is a four-channel EEG monitoring device that collects data from the frontal lobe of the brain at locations specified through Fp1, Fp2, F7, and F8; it has been developed to monitor sedation depth and brain electrical activity in patients who are under anesthesia. Electrode impedance was maintained at less than 5 kΩ in each channel. The EEG was recorded continuously from the baseline (before anesthesia) to the end of surgery. The EEG results were independently analyzed with a focus on amplitude and frequency according to the American Clinical Neurophysiology Society’s Standardized Critical Care EEG Terminology (14) and classified as rhythmic polyspikes (PSR), periodic epileptiform discharges (PED), delta with spikes (DSP), and suppression with spikes (SSP) (15). The EEG was interpreted by neuroelectrophysiology physicians. The data were considered as epileptiform discharges based on the agreement of neuroelectrophysiology physicians.

### Data collection

Data regarding the patient’s general condition, history of alcohol use, and comorbid conditions were recorded. The baseline cognitive function was evaluated using the Mini-Mental State Examination (MMSE). The time of intraoperative surgery and details of anesthesia including the dosage of anesthesia and vasoactive drugs used were also recorded. The hemoglobin after surgery, LOS, and other complications (cardiac events, cerebrovascular events, kidney injury, and infection, among others) that occurred during the postoperative hospitalization were also recorded.

### Postoperative delirium assessment and analysis

The development of delirium was the primary outcome of the study. The presence of delirium was evaluated by trained research team members using the Confusion Assessment Method (CAM) or the Confusion Assessment Method for the Intensive Care Unit (CAM-ICU) at specific times, as their reliability has been previously demonstrated (16). Researchers screened patients for delirium using the CAM or the CAM-ICU twice a day (at 6–9 am and 6–9 pm) for 3 days, starting from the first day after surgery. The CAM-ICU was used to evaluate those patients who had a Richmond Agitation Sedation Scale score of −3 or greater during cardiac surgical care unit admission with stable hemodynamics and respiration and in whom the tracheal tube could not be removed or who could not answer questions. Patients who did not achieve a Richmond Agitation Sedation Scale score of −3 were reassessed later, and the score was recorded. Patients were classified as having postoperative delirium if they screened positive at any time.

### Sample size calculation

The sample size was calculated based on the incidence of postoperative delirium in a previous study, where 72% and 32% of patients undergoing surgery did and did not have epileptiform discharges, respectively (17). The sample size that would yield a statistical power of 90% at the difference based on a two-tailed significance level of 0.05 was calculated to be 62 patients. Considering an estimated dropout rate of 20%, we intended to enroll 76 patients. The sample size was calculated using PASS 11.0 software.

### Statistical analysis

Statistical analysis was performed using SPSS 25.0 software. Data are presented as medians with the interquartile range (IQR) or as frequencies (*n*) and percentages (%). Significant differences in categorical variables were compared between the groups using the chi-square test. For parametric continuous variables, the *t*-test was used. Continuous variables that did not meet the criteria for parametric testing were evaluated using the Mann–Whitney *U* test. The odds ratio with 95% confidence intervals and corresponding *P* values were calculated for each risk factor. To determine the impact of epileptiform discharges on the incidence of delirium, we performed a binary logistic regression analysis of independent associations. Variables that significantly affected delirium on univariate analysis were included during multivariate logistic regression analysis. A two-sided *P* value of <0.05 was considered statistically significant.

## Results

**Figure 1:**
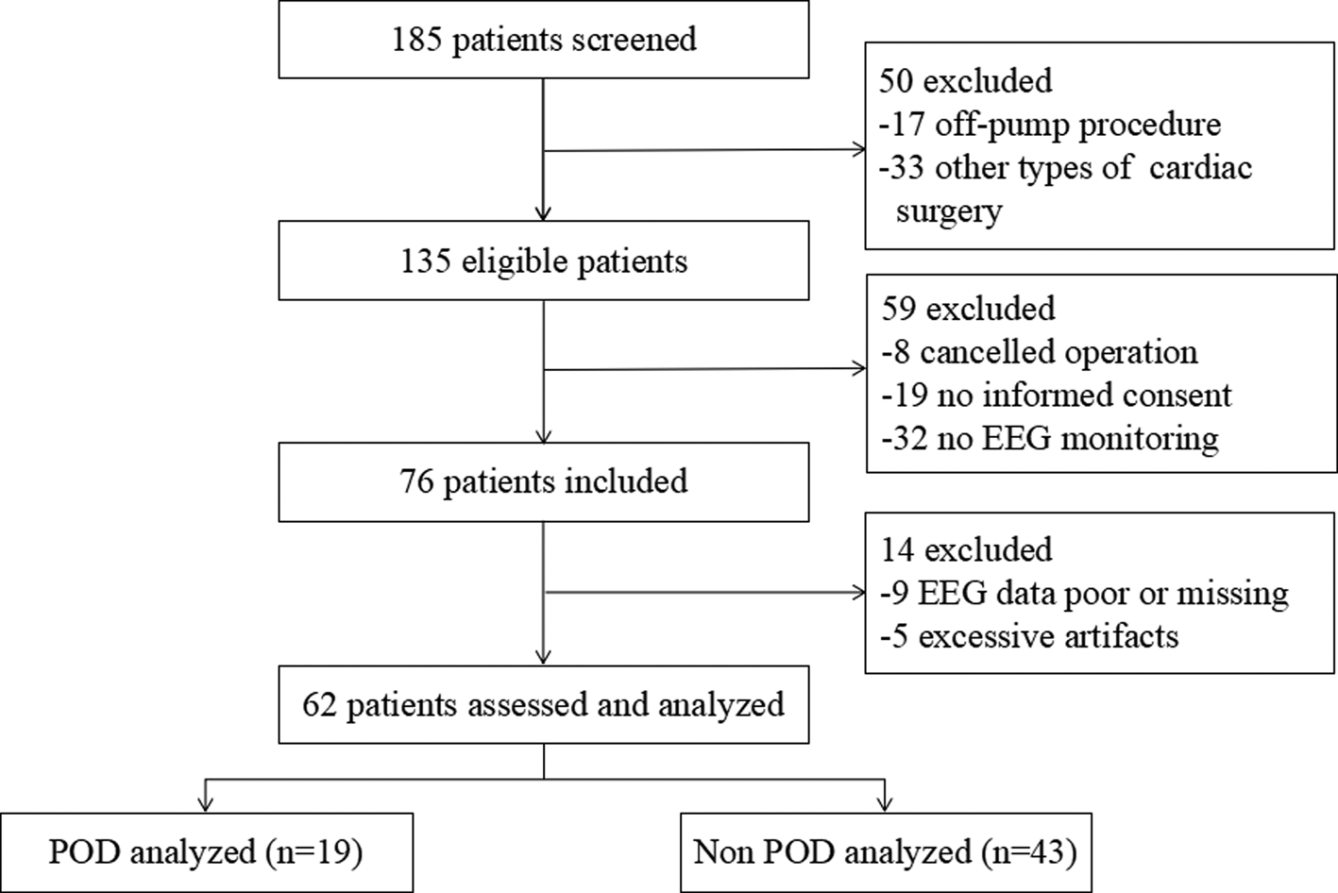
Flow diagram demonstrating the process of patient enrollment. POD, postoperative delirium; EEG, electroencephalogram.

The study flow diagram is presented in Figure 1. Patients were enrolled between July 2, 2020, and July 16, 2021. A total of 76 patients were selected for this prospective observational study, of whom 62 were eventually enrolled and their data analyzed. The intraoperative EEG data could not be analyzed in nine and five patients owing to poor or incomplete data collection during surgery and excessive artifacts (eyelid motion and muscle artifacts), respectively.

The median age of the remaining patients was 59 years; 65% were male, and the median body mass index was 23.95 kg/m^2^. At presentation, the physical status of all patients was of grade III, according to the American Society of Anesthesiologists. The MMSE scale was used to assess the preoperative cognitive function of the patients; the median value was found to be 25. The median anesthesia maintenance, CPB, and aortic cross-clamp times were 330, 135, and 88 min, respectively. On postoperative delirium screening (CAM or CAM-ICU), 19/62 (31%) patients tested positive; 43/62 (69%) did not have postoperative delirium. The characteristics of the patients in the two groups are summarized in Table 1; there were no significant differences between the two groups in terms of baseline characteristics (*P* > 0.05). Patients in the delirium group differed from those in the nondelirium group in that they were older [64 (60–66) vs. 56 (51–62) years, *P* = 0.001] and the LOS was longer [21 (17–27) vs. 18 (13–22) days, *P* = 0.004]; anesthesia (*P* = 0.023) and cardiopulmonary bypass (*P* = 0.015) times were also longer; and hemoglobin after surgery was lower [100 (113–129) vs. 120 (132–148), *P* = 0.001].

**Table 1:** Comparison between patients with and without delirium.

	POD (*n* = 19)		Non-POD (*n* = 43)		*P* value
	Median	[IQR]	Median	[IQR]	
Age, years	64	[60–66]	56	[51–62]	0.001
Height, cm	165	[160–170]	169	[160–171]	0.857
Weight, kg	66	[59–76]	65	[60–75]	0.937
BMI, kg/m^2^	23.74	[22.04–25.35]	24.06	[22.39–25.80]	0.982
Sex, male/female	11/8		29/14		0.469
Preoperative medications					
Aspirin	1 (5.30%)		2 (4.65%)		0.918
ACE inhibitor	2 (10.53%)		7 (16.28%)		0.553
ARB agent	6 (31.58%)		15 (34.88%)		0.800
*β*-blocker	1 (5.30%)		8 (18.60%)		0.169
Calcium channel blocker	2 (10.53%)		8 (18.60%)		0.425
Hemoglobin, g/dl	134	[144–151]	136	[148–158]	0.369
Comorbid conditions					
Hypertension	8 (42.11%)		13 (30.23%)		0.362
Atrial fibrillation	3 (15.79%)		4 (9.30%)		0.457
Coronary heart disease	2 (10.53%)		4 (9.30%)		0.881
Diabetes	1 (5.26%)		1 (2.33%)		0.546
Alcohol use	1 (5.26%)		4 (9.30%)		0.590
Epileptiform discharges	10 (52.63%)		6 (13.95%)		0.001
Procedures					
Operation duration, min	320	[229–441]	260	[212–291]	0.018
Anesthesia duration, min	375	[295–491]	313	[272–352]	0.023
CPB duration, min	184	[126–214]	120	[95–155]	0.015
Aortic cross-clamp, min	115	[55–159]	80	[50–110]	0.067
Drug					
Propofol, mg	950	[720–1300]	767	[625–1059]	0.089
Remifentanil, mg	3.72	[2.68–4.78]	3.57	[2.76–4.36]	0.487
Dopamine, mg	47.60	[26.15–97.50]	34.65	[19.60–49.00]	0.040
Epinephrine, µg	140.00	[0.00–220.00]	142.50	[27.70–274.00]	0.596
Nitroglycerin, mg	0.69	[0.51–1.01]	0.96	[0.54–1.90]	0.141
Atropine	5 (26.32%)		4 (9.30%)		0.080
Hemoglobin after surgery, g/dl	100	[113–129]	120	[132–148]	0.001
Red cell transfusion	5 (26.32%)		1 (2.33%)		0.003
Length of stay	21	[17–27]	17	[13–22]	0.004
Preoperative MMSE	24	[23–25]	26	[22–27]	0.146
Complication, *n* (%)	7 (36.84%)		11 (25.58%)		0.368

Values are presented as medians and IQR or frequencies (n) and percentages (%). Statistical analysis was performed with the t-test, Mann–Whitney U test, or Pearson chi-square test.

POD, postoperative delirium; BMI, body mass index; CPB, cardiopulmonary bypass; MMSE, Mini-Mental Status Examination; ACE, angiotensin-converting enzyme; ARB, angiotensin receptor blocker; IQR, interquartile range.

Epileptiform discharges occurred in 16/62 (26%) of the patients. Also, epileptiform discharges were more common in patients with delirium (62.50% vs. 19.57%, *P* < 0.001). As shown in Table 2, there were no significant differences in baseline characteristics between the groups with and without epileptiform discharges (*P* > 0.05). In addition, the hemoglobin level after surgery also showed no significant differences between the two groups (*P* > 0.05). The LOS was longer in the group with epileptiform discharges [21 (18–28) vs. 18 (14–22) days, *P* = 0.031]. Epileptiform discharges occurred in 5 and 12 patients during anesthesia induction and CPB, respectively. In all patients with epileptiform discharges, DSP discharges are the most common in eight (50%) of the cases, six (38%) of PSR, three (19%) of PED, and one (6%) of SSP.

**Table 2:** Comparison between patients with and without epileptiform discharges.

	Epileptiform discharges (*n* = 16)		Non-epileptiform discharges (*n* = 46)		*P* value
	Median	[IQR]	Median	[IQR]	
Age, years	63	[59–67]	57	[51–64]	0.079
Height, cm	166	[160–169]	169	[160–171]	0.767
Weigh, kg	66	[58–70]	66	[60–76]	0.509
BMI, kg/m^2^	23.04	[22.04–24.81]	24.22	[22.47–25.89]	0.145
Sex, male/female	11/5		29/17		0.681
Delirium	10 (62.50%)		9 (19.57%)		0.001
Hemoglobin, g/dl	129	[146–155]	137	[147–156]	0.299
Procedures					
Operation duration, min	270	[197–320]	271	[221–340]	0.647
Anesthesia duration, min	333	[250–371]	330	[290–397]	0.595
CPB duration, min	136	[76–180]	133	[104–186]	0.573
Aortic cross-clamp, min	84	[42–128]	89	[63–124]	0.359
Drug					
Propofol, mg	899	[679–1078]	778	[650–1074]	0.494
Remifentanil, mg	3.43	[2.59–4.47]	3.76	[2.78–4.58]	0.479
Dopamine, mg	41.28	[27.33–55.38]	38.25	[21.21–67.00]	0.687
Epinephrine, µg	131.98	[26.79–212.25]	150.50	[0.00–304.90]	0.722
Nitroglycerin, mg	0.89	[0.56–1.46]	0.91	[0.51–1.94]	0.904
Hemoglobin after surgery, g/dl	104	[129–136]	116	[127–145]	0.519
Length of stay	21	[18–28]	18	[14–22]	0.031
Preoperative MMSE	24	[22–26]	26	[23–27]	0.083

Values are presented as medians and IQR or frequencies (n) and percentages (%). Statistical analysis was performed with the t-test, Mann–Whitney U test, or Pearson chi-square test.

BMI, body mass index; CPB, cardiopulmonary bypass; MMSE, Mini-Mental Status Examination; IQR, interquartile range.

A total of 18 patients developed postoperative complications; patients with delirium had higher complication rates than those without delirium (36.84% vs. 25.58%). However, it was unclear whether ischemic or latent stroke occurred, as confirmatory computed tomography or magnetic resonance imaging was not performed. In addition, none of the patients admitted to the cardiac surgical intensive care unit were observed to have seizures.

### Univariate analyses of independent associations

For univariate binary logistic regression analysis, the occurrence of delirium was used as the dependent variable, and the factors associated with delirium were used as independent variables. The preoperative MMSE score did not differ between patients with and without delirium (*P* = 0.153). Likewise, the duration of surgery and anesthesia and dosage of anesthesia did not differ between the groups (*P* = 0.122, *P* = 0.754, and *P* = 0.273, respectively). Previous studies have shown that prolonged CPB leads to poor prognosis in patients undergoing cardiac surgery; however, there was no statistically significant difference between the two groups in this study [odds ratio, 2.70 (0.77–9.50); *P* = 0.122]. Univariate analysis showed that only age [odds ratio, 6.33 (1.79–22.41); *P* = 0.004] and epileptiform discharges [odds ratio, 6.85 (1.97–23.84); *P* = 0.002] were significantly and independently associated with postoperative delirium in our study (Table 3). The incidence of delirium increased with age; 52% of patients were aged over 60 years. In this study, only age was found to be related to epileptiform discharges [odds ratio, 4.26 (1.19–15.25); *P* = 0.026]. We did not find other factors, such as time of CPB and dose of anesthetic drugs, to be related to epileptiform discharges.

**Table 3:** Univariate and multivariate logistic regression analyses for prediction of postoperative delirium in patients after surgery.

	Univariable		Multivariable	
	Odds ratio (95% CI)	*P* value	Odds ratio (95% CI)	*P* value
Age	6.33 (1.79–22.41)	0.004	4.75 (1.26–17.92)	0.022
Operation duration	2.70 (0.77–9.50)	0.122	—	—
Anesthesia duration	1.21 (0.36–4.07)	0.754	—	—
CPB duration	2.70 (0.77–9.50)	0.122	—	—
Aortic cross-clamp duration	1.08 (0.32–3.67)	0.897	—	—
Propofol	1.88 (0.61–5.81)	0.273	—	—
Remifentanil	1.21 (0.36–4.07)	0.754	—	—
Preoperative MMSE	0.85 (0.69–1.06)	0.153	—	—
Epileptiform discharges	6.85 (1.97–23.84)	0.002	5.00 (1.34–18.74)	0.017

The results of logistic regression analysis have been presented as the odds ratio with 95% confidence intervals.

CPB, cardiopulmonary bypass; MMSE, Mini-Mental Status Examination; CI, confidence interval.

### Multivariable logistic regression analyses

On univariate logistic regression analysis of the risk factors for delirium, only age and epileptiform discharges were considered relevant predictors for further multivariate logistic regression analysis. The overall model for both predictors was significant. The values were as follows: age [odds ratio, 4.75 (1.26–17.92), *P* = 0.022] and epileptiform discharges [odds ratio, 5.00 (1.34–18.74), *P* = 0.017] (Table 3).

## Discussion

In this study, we found that the incidence of epileptiform discharges is surprisingly high in patients undergoing cardiac surgery; they were observed in 16/62 (26%) of patients. Overall, 19/62 (31%) of patients developed delirium after cardiac surgery. Epileptiform discharges and age were identified as independent confounders related to the development of delirium in patients after cardiac surgery. Therefore, we speculate that the occurrence of epileptiform discharges is related to postoperative delirium in patients undergoing cardiac surgery.

We found that among the patients who are at a high risk of delirium after cardiac surgery, approximately 31% developed delirium; this agrees with the incidence of delirium (23%–52%) reported by previous studies on patients who underwent cardiac surgery (18, 19). Although the pathophysiological mechanism of delirium remains unclear, the changes in brain electrical activity caused by a variety of factors play an important role in pathogenesis; these include neurotransmitter imbalance, changes in neuronal excitability, and overactivated inflammatory responses (20, 21). In particular, neurotransmitter imbalances such as increased dopamine and glutamate and decreased glutamine in the cerebrospinal fluid may increase the fragility of the brain, thereby contributing to delirium (22).

EEG is an effective tool for monitoring the electrical activity of the brain. Epileptiform discharges are characterized by abnormal spontaneous discharges in the brain that can affect cognition and awareness. Hanak et al. (23) found that the expression of metabolic glutamate receptor 5 in the hippocampus decreases during epileptic seizures; this leads to a large accumulation of glutamate, which acts on ionic receptors. This in turn results in Ca^2+^ and Na^+^ influx and K^+^ outflow, inducing abnormal synchronous discharge and epileptic seizures. Inflammatory processes in the brain and damage to the blood–brain barrier often cause destruction of ion channels, abnormal neurotransmitter uptake and release, and excitatory neurotoxicity (24). This indicates that the occurrence of epileptiform discharges and delirium may have a common mechanism. Epileptiform discharges can be observed in EEG records of other surgical patients, especially in those with brain dysfunction (25). In addition, epileptiform discharges can also occur in patients without seizures or a diagnosis of epilepsy (26). In our study, the intensive care unit staff did not observe seizures in the patients after surgery.

In our study, 26% of patients demonstrated epileptiform discharges during cardiac surgery. In another study on postoperative patients in the cardiac surgical care unit, Tschernatsch et al. (27) found the incidence of epileptiform discharges and abnormal EEGs to be 9% and 33%, respectively. The high incidence of epileptiform discharges in our study may be attributed to the fact that we analyzed intraoperative EEG data and abnormal electrical activity in the brain caused by anesthesia drugs and changes in cerebral blood flow during CPB. Our study found epileptiform discharges to occur during anesthesia induction in 5/16 (31%) patients. A previous study found that anesthesia drugs, such as propofol and sevoflurane, can induce delirium and epileptiform discharges (28). Therefore, the sudden increase in blood concentration of propofol and other anesthesia drugs during anesthesia induction may cause abnormal brain discharges and be related to the occurrence of epileptiform discharges. In addition, 12/16 (75%) patients had epileptiform discharges during CPB, which may be because of ischemic hypoxia, hypoperfusion, and hyperperfusion of the brain tissue during CPB and cause ion channel and blood–brain barrier dysfunction, leading to abnormal neuronal firing and seizures (29, 30). Therefore, the occurrence of epileptiform discharges on intraoperative EEGs may be related to the above factors; however, its specific mechanism needs further exploration.

Previous studies have shown that EEG may correlate with the occurrence of delirium (31, 32). Fritz et al. (33) found that a longer duration of intraoperative EEG suppression is associated with a higher occurrence of delirium. Eskioglou et al. (34) found that the EEG of patients with delirium in the intensive care unit may demonstrate burst suppression, rhythmic or periodic patterns, and seizures or status epilepticus. Patients with delirium also show increased slow-wave activity involving the occipitoparietal and frontal cortex with disruption of functional connectivity (35). These findings imply that EEGs play an important role in predicting the occurrence of delirium.

This study further explored and analyzed the relationship between intraoperative EEG and delirium. Using univariate and multivariate logistic regression analysis models, we found a significant association between epileptiform discharges and delirium. In our study, we found that epileptiform discharges were more common in patients with delirium. The results of the univariate logistic analysis showed that the occurrence of epileptiform discharges and age were independent confounding factors for delirium.

In this context, persistent cognitive deficits in terms of memory and learning occur in patients with epileptic seizures or persistent epileptic states (36) and the use of the antiepileptic drug levetiracetam can effectively reverse behavioral abnormalities and reduce epileptic seizures in patients with Alzheimer’s disease (37). Therefore, the identification and detection of early epilepsy and appropriate timely treatment may have important clinical implications for the occurrence and development of delirium. The results from the present study provide a valuable basis for further study of the sources and clinical effects of abnormal EEG discharges.

Age is currently known to be a risk factor for delirium. Our study found a significant association between age and delirium; the incidence of delirium increased with age. The two groups in our study did not differ in terms of other risk factors for delirium, including a history of alcohol use and comorbid conditions; we found that the LOS was longer in patients with delirium.

The results of this study offer promise for the diagnosis of delirium. However, certain limitations need to be mentioned. First, the patients were only monitored through intraoperative EEG; they were not monitored after admission to the cardiac surgical care unit and the staff did not record any convulsions. Second, we excluded patients with cognitive impairment and dementia before surgery; this may make our study less comprehensive. Therefore, the results cannot be generalized to patients with cognitive dysfunction to assess whether the preoperative cognitive state has any relation with intraoperative epileptiform discharges and postoperative delirium. Third, the anesthesia drugs may affect EEG electrical activity. Fourth, we did not analyze the duration of burst suppression, other abnormal brain waves, and the duration of epileptiform discharges. At last, we did not have access to tools such as computed tomography and magnetic resonance imaging to confirm the occurrence of stroke, cerebral microthrombosis, and other cerebrovascular accidents after surgery. In the future, it will be necessary to combine EEG recordings with findings on magnetic resonance imaging or other investigations to establish the association between epileptiform discharges and delirium.

## Conclusion

Our results suggest that intraoperative epileptiform discharges on EEG during cardiac surgery may be associated with the occurrence of postoperative delirium. The results of this study indicate the need for additional research to further characterize epileptiform discharges, investigate whether interventions can reduce the incidence of delirium, and determine any causal relationships between intraoperative EEG abnormalities and postoperative delirium.

## Data Availability

The original contributions presented in the study are included in the article/Supplementary Material, further inquiries can be directed to the corresponding author.

## References

[1] Aldecoa C Bettelli G Bilotta F Sanders RD Audisio R Borozdina A European Society of Anaesthesiology evidence-based and consensus-based guideline on postoperative delirium. Eur J Anaesthesiol. (2017) 34:192–214. 10.1097/EJA.0000000000000594 28187050

[2] Rudolph JL Jones RN Levkoff SE Rockett C Inouye SK Sellke FW Derivation and validation of a preoperative prediction rule for delirium after cardiac surgery. Circulation. (2009) 119(2):229–36. 10.1161/CIRCULATIONAHA.108.795260 19118253 PMC2735244

[3] Klein Klouwenberg PM Zaal IJ Spitoni C Ong DS van der Kooi AW Bonten MJ The attributable mortality of delirium in critically ill patients: prospective cohort study. Br Med J. (2014) 349:g6652. 10.1136/bmj.g6652 25422275 PMC4243039

[4] Marcantonio ER. Postoperative delirium: a 76-year-old woman with delirium following surgery. JAMA. (2012) 308:73–81. 10.1001/jama.2012.6857 22669559 PMC3604975

[5] Glumac S Kardum G Karanovic N. Postoperative cognitive decline after cardiac surgery: a narrative review of current knowledge in 2019. Med Sci Monit. (2019) 25:3262–70. 10.12659/MSM.914435 31048667 PMC6511113

[6] Sauër AC Veldhuijzen DS Ottens TH Slooter AJC Kalkman CJ van Dijk D. Association between delirium and cognitive change after cardiac surgery. Br J Anaesth. (2017) 119:308–15. 10.1093/bja/aex053 28854542

[7] Zaal IJ Slooter AJ. Delirium in critically ill patients: epidemiology, pathophysiology, diagnosis and management. Drugs. (2012) 72:1457–71. 10.2165/11635520-000000000-00000 22804788

[8] Mulkey MA Hardin SR Olson DM Munro CL. Pathophysiology review: seven neurotransmitters associated with delirium. Clin Nurse Spec. (2018) 32:195–211. 10.1097/NUR.0000000000000384 29878931

[9] Goldstone AB Bronster DJ Anyanwu AC Goldstein MA Filsoufi F Adams DH Predictors and outcomes of seizures after cardiac surgery: a multivariable analysis of 2,578 patients. Ann Thorac Surg. (2011) 91:514–8. 10.1016/j.athoracsur.2010.10.090 21256303

[10] Ibrahim GM Cassel D Morgan BR Smith ML Otsubo H Ochi A Resilience of developing brain networks to interictal epileptiform discharges is associated with cognitive outcome. Brain. (2014) 137:2690–702. 10.1093/brain/awu214 25104094 PMC4163036

[11] Lam AD Sarkis RA Pellerin KR Jing J Dworetzky BA Hoch DB Association of epileptiform abnormalities and seizures in Alzheimer disease. Neurology. (2020) 95:e2259–70. 10.1212/WNL.0000000000010612 32764101 PMC7713786

[12] You KM Suh GJ Kwon WY Kim KS Ko SB Park MJ Epileptiform discharge detection with the 4-channel frontal electroencephalography during post-resuscitation care. Resuscitation. (2017) 117:8–13. 10.1016/j.resuscitation.2017.05.016 28511986

[13] Luz-Escamilla L Morales-González JA. Association between interictal epileptiform discharges and autistic spectrum disorder. Brain Sci. (2019) 9:185. 10.3390/brainsci9080185 PMC6721430 31366163

[14] Hirsch LJ Fong MWK Leitinger M LaRoche SM Beniczky S Abend NS American clinical neurophysiology society's standardized critical care EEG terminology: 2021 version. J Clin Neurophysiol. (2021) 38:1–29. 10.1097/WNP.0000000000000806 33475321 PMC8135051

[15] Kane N Acharya J Benickzy S Caboclo L Finnigan S Kaplan PW A revised glossary of terms most commonly used by clinical electroencephalographers and updated proposal for the report format of the EEG findings. Revision 2017. Clin Neurophysiol Pract. (2017) 2:170–85. 10.1016/j.cnp.2017.07.002 30214992 PMC6123891

[16] Maybrier HR Mickle AM Escallier KE Lin N Schmitt EM Upadhyayula RT Reliability and accuracy of delirium assessments among investigators at multiple international centres. BMJ Open. (2018) 8:e023137. 10.1136/bmjopen-2018-023137 30467132 PMC6252643

[17] Koch S Rupp L Prager C Wernecke KD Kramer S Fahlenkamp A Emergence delirium in children is related to epileptiform discharges during anaesthesia induction: an observational study. Eur J Anaesthesiol. (2018) 35:929–36. 10.1097/EJA.0000000000000867 30113351

[18] Wildes TS Mickle AM Ben Abdallah A Maybrier HR Oberhaus J Budelier TP Effect of electroencephalography-guided anesthetic administration on postoperative delirium among older adults undergoing major surgery: the ENGAGES randomized clinical trial. JAMA. (2019) 321:473–83. 10.1001/jama.2018.22005 30721296 PMC6439616

[19] Rudolph JL Jones RN Levkoff SE Rockett C Inouye SK Sellke FW Derivation and validation of a preoperative prediction rule for delirium after cardiac surgery. Circulation. (2009) 119:229–36. 10.1161/CIRCULATIONAHA.108.795260 19118253 PMC2735244

[20] Matsumoto Y Fujino Y Furue H. Anesthesia and surgery induce a functional decrease in excitatory synaptic transmission in prefrontal cortex neurons, and intraoperative administration of dexmedetomidine does not elicit the synaptic dysfunction. Biochem Biophys Res Commun. (2021) 572:27–34. 10.1016/j.bbrc.2021.07.065 34332326

[21] Kotfis K Ślozowska J Safranow K Szylińska A Listewnik M. The practical use of white cell inflammatory biomarkers in prediction of postoperative delirium after cardiac surgery. Brain Sci. (2019) 9:308. 10.3390/brainsci9110308 PMC6896029 31684066

[22] Han Y Zhang W Liu J Song Y Liu T Li Z Metabolomic and lipidomic profiling of preoperative CSF in elderly hip fracture patients with postoperative delirium. Front Aging Neurosci. (2020) 12:570210. 10.3389/fnagi.2020.570210 33192460 PMC7642614

[23] Hanak TJ Libbey JE Doty DJ Sim JT DePaula-Silva AB Fujinami RS. Positive modulation of mGluR5 attenuates seizures and reduces TNF-α+ macrophages and microglia in the brain in a murine model of virus-induced temporal lobe epilepsy. Exp Neurol. (2019) 311:194–204. 10.1016/j.expneurol.2018.10.006 30316834 PMC6263825

[24] Dey A Kang X Qiu J Du Y Jiang J. Anti-inflammatory small molecules to treat seizures and epilepsy: from bench to bedside. Trends Pharmacol Sci. (2016) 37:463–84. 10.1016/j.tips.2016.03.001 27062228 PMC5064857

[25] Kurtz P Gaspard N Wahl AS Bauer RM Hirsch LJ Wunsch H Continuous electroencephalography in a surgical intensive care unit. Intensive Care Med. (2014) 40:228–34. 10.1007/s00134-013-3149-8 24240843

[26] Vossel KA Ranasinghe KG Beagle AJ Mizuiri D Honma SM Dowling AF Incidence and impact of subclinical epileptiform activity in Alzheimer's disease. Ann Neurol. (2016) 80:858–70. 10.1002/ana.24794 27696483 PMC5177487

[27] Tschernatsch M Juenemann M Alhaidar F El Shazly J Butz M Meyer M Epileptic seizure discharges in patients after open chamber cardiac surgery-a prospective prevalence pilot study using continuous electroencephalography. Intensive Care Med. (2020) 46(7):1418–24. 10.1007/s00134-020-06073-8 32405742 PMC7334279

[28] Koch S Rupp L Prager C Mörgeli R Kramer S Wernecke KD Incidence of epileptiform discharges in children during induction of anaesthesia using propofol versus sevoflurane. Clin Neurophysiol. (2018) 129:1642–8. 10.1016/j.clinph.2018.05.013 29913339

[29] Zayachkivsky A Lehmkuhle MJ Ekstrand JJ Dudek FE. Ischemic injury suppresses hypoxia-induced electrographic seizures and the background EEG in a rat model of perinatal hypoxic-ischemic encephalopathy. J Neurophysiol. (2015) 114:2753–63. 10.1152/jn.00796.2014 26354320 PMC4644230

[30] Leonhardt G de Greiff A Weber J Ludwig T Wiedemayer H Forsting M Brain perfusion following single seizures. Epilepsia. (2005) 46:1943–9. 10.1111/j.1528-1167.2005.00336.x 16393160

[31] Numan T van den Boogaard M Kamper AM Rood PJT Peelen LM Slooter AJC. Delirium detection using relative delta power based on 1-minute single-channel EEG: a multicentre study. Br J Anaesth. (2019) 122:60–8. 10.1016/j.bja.2018.08.021 30579407

[32] Ma O Crepeau AZ Dutta A Bliss DW. Anticipating postoperative delirium during burst suppression using electroencephalography. IEEE Trans Biomed Eng. (2020) 67:2659–68. 10.1109/TBME.2020.2967693 32031924

[33] Fritz BA Maybrier HR Avidan MS. Intraoperative electroencephalogram suppression at lower volatile anaesthetic concentrations predicts postoperative delirium occurring in the intensive care unit. Br J Anaesth. (2018) 121:241–8. 10.1016/j.bja.2017.10.024 29935578 PMC6200110

[34] Eskioglou E Iaquaniello C Alvarez V Rüegg S Schindler K Rossetti AO Electroencephalography of mechanically ventilated patients at high risk of delirium. Acta Neurol Scand. (2021) 144:296–302. 10.1111/ane.13447 33950516 PMC8453526

[35] Tanabe S Mohanty R Lindroth H Casey C Ballweg T Farahbakhsh Z Cohort study into the neural correlates of postoperative delirium: the role of connectivity and slow-wave activity. Br J Anaesth. (2020) 125:55–66. 10.1016/j.bja.2020.02.027 32499013 PMC7339874

[36] Vossel KA Tartaglia MC Nygaard HB Zeman AZ Miller BL. Epileptic activity in Alzheimer's disease: causes and clinical relevance. Lancet Neurol. (2017) 16:311–22. 10.1016/S1474-4422(17)30044-3 28327340 PMC5973551

[37] Xu Y Lavrencic L Radford K Booth A Yoshimura S Anstey KJ Systematic review of coexistent epileptic seizures and Alzheimer's disease: incidence and prevalence. J Am Geriatr Soc. (2021) 69:2011–20. 10.1111/jgs.17101 33740274

